# Clinical management of feline chronic kidney disease in Portugal: a questionnaire-based study

**DOI:** 10.1177/1098612X231206125

**Published:** 2023-11-21

**Authors:** Tomás Rodrigues Magalhães, Ana Luísa Lourenço, Ronald Jan Corbee, Felisbina Luísa Queiroga

**Affiliations:** 1 Department of Veterinary Sciences, University of Trás-os-Montes and Alto Douro, Vila Real, Portugal; 2 Animal and Veterinary Research Centre (CECAV), University of Trás-os-Montes and Alto Douro, Vila Real, Portugal; 3 Associate Laboratory for Animal and Veterinary Sciences (AL4AnimalS), University of Trás-os-Montes and Alto Douro, Vila Real, Portugal; 4 Department of Animal Science, University of Trás-os-Montes and Alto Douro, Vila Real, Portugal; 5 Department of Clinical Sciences, Faculty of Veterinary Medicine, Utrecht University, Utrecht, The Netherlands; 6 Centre for the Study of Animal Science, CECA-ICETA, University of Porto, Porto, Portugal

**Keywords:** Chronic kidney disease, IRIS, questionnaire-based study, renal disease

## Abstract

**Objectives:**

The aim of the study was to characterise the clinical management of feline chronic kidney disease (CKD) by veterinary practitioners in Portugal.

**Methods:**

A questionnaire-based study was designed to be completed by all veterinarians who had diagnosed and treated at least one case of feline CKD in the previous year.

**Results:**

A total of 409 veterinary practitioners responded to the questionnaire, with approximately half of them diagnosing 2–5 cases of feline CKD per month (n = 219, 53.5%). Although a high proportion of these reported using the guidelines published by the International Renal Interest Society (n = 379, 92.7%), only 19.1% (n = 78) systematically performed systolic blood pressure (SBP) measurements in all of their patients. A renal diet was advised by almost all respondents (n = 406, 99.3%), but 36.9% (n = 150) of them considered that it represented less than 75% of the daily food intake for most of their patients. This dietary intervention was often prescribed regardless of stage (n = 298, 73.4%) and without a proper gradual diet transition. Appetite stimulants were frequently prescribed (n = 366, 89.5%), as well as a calcium channel blocker (n = 171, 41.8%) and an angiotensin-converting enzyme inhibitor (n = 245, 59.9%) to control systemic hypertension and proteinuria, respectively. Prescription of a phosphate binder was also common (n = 311, 76.0%). Regarding monitoring, 70.9% (n = 290) recommended that stable patients be reassessed every 2–3 months or more frequently, but only 35.7% (n = 146) were able to comply with this periodicity due to owners’ constraints.

**Conclusions and relevance:**

The findings showed that although most survey respondents are aware of international guidelines for the clinical management of cats with CKD, the SBP measurement still needs to be more systematic to allow proper substaging and detection of systemic hypertension. The monitoring frequency was lower than recommended. Furthermore, the introduction of a renal therapeutic diet should be refined to improve its acceptance rate.

## Introduction

Chronic kidney disease (CKD) has been considered one of the most commonly diagnosed metabolic disorders in feline medicine, particularly in middle-aged to senior cats, with an estimated overall prevalence in the range of 1.2–50%, according to different cat populations.^1–4^ A major clinical impact on this domestic species has been reported given the high morbidity and mortality associated with this disease, which tends to increase with age.^3,5,6^ In fact, O’Neill et al^7^ described ‘renal disorder’ as the most frequent cause of death (13.6%) in cats aged above 5 years.

The diagnosis of CKD can be achieved through the combination of clinical and laboratory findings compatible with impairment of renal function: increased serum concentrations of creatinine and/or symmetric dimethylarginine (SDMA); an inappropriately low urine specific gravity (USG); suggestive ultrasound findings, such as loss of corticomedullary distinction; and/or clinical signs compatible with the disease, such as anorexia, weight loss and polyuria/polydipsia.^8,9^ Regarding the two serum biomarkers (creatinine and SDMA), both are associated with glomerular filtration rate (GFR), but differ in their sensitivity. SDMA rises when there is on average approximately a 40% decrease in GFR, while creatinine shows an increase only when this percentage reduction is already 75%.^10,11^ Thus, SDMA has been considered a more sensitive kidney biomarker, allowing earlier detection of CKD in the feline population.^12^

After the diagnosis is established, the clinician must perform disease staging and substaging. According to the International Renal Interest Society (IRIS) guidelines, feline CKD should be staged according to fasting serum creatinine and/or SDMA concentrations measured in a stable and well-hydrated patient, on at least two time points.^11^ Subsequently, substaging can be performed based on the evaluation of proteinuria, considering the urinary protein-to-creatinine ratio (UPCR), ideally assessed on at least two urine samples obtained at least 2 weeks apart, and the measurement of systolic blood pressure (SBP).

Therapeutic and management strategies should be implemented based on disease stage and substage in order to delay progression of the disease, to preserve the remaining renal function, to control the clinical signs and to guarantee the patient’s quality of life.^13^ Dietary intervention and optimised hydration are the cornerstones of treatment of CKD. However, clinical sequelae to CKD are common in these patients, including proteinuria, hypertension, hyperphosphataemia as part of CKD-mineral and bone disorder, anaemia and metabolic acidosis. Therefore, a carefully planned and regularly adjusted approach to management is usually required.^8,14^ This should be individualised to each cat diagnosed with CKD and may include one or more of the following products/drugs, each with a specific therapeutic goal: antiemetics and appetite stimulants to relieve clinical signs; potassium supplements and phosphate binders to correct electrolyte disorders; angiotensin-converting enzyme (ACE) inhibitors, angiotensin receptor blockers (ARBs) and calcium channel blockers (CCBs) to control proteinuria and/or hypertension; and erythrocyte-stimulating agents to treat anaemia.^8,13^ Moreover, it should be noted that the management of feline CKD can be challenging, since therapeutic success depends on the compliance of the owner and the patient, which in these cases may be limited.^9,15,16^

The aim of this questionnaire-based study was to characterise the clinical management of feline CKD by veterinary practitioners in Portugal, particularly regarding its diagnosis, treatment and monitoring.

## Materials and methods

A questionnaire was designed consisting of 29 closed questions. The questions were either multiple choice or checkboxes, in which respondents could choose only one or more than one answer, respectively. At the end of the questionnaire, there was also an open question for additional comments. The answers to this last question were not intended to be used in the current study, but rather to identify other aspects of the clinical management of feline CKD that were not addressed in the questionnaire and that could be of potential interest for further studies. Before being publicly shared, the questionnaire underwent two validation phases. Initially, a first version of the survey was evaluated by an epidemiologist. After approval, it was sent to 10 veterinary practitioners. The group of veterinary practitioners consisted of individuals from several geographical regions and age groups with different professional experience. Their responses were considered only in order to detect possible errors or difficulties in understanding the questionnaire; therefore, they were not included in the analysis. After the validation process, the questionnaire was improved, and the final version was uploaded to an online platform (Google Forms). The link was sent to all existing veterinary clinics and hospitals in Portugal (mainland and autonomous regions) based on the list available on the Directorate-General for Food and Veterinary Medicine website (https://www.dgav.pt, accessed on 17 July 2022). In addition, the questionnaire was disseminated to social media groups that were exclusive to Portuguese veterinarians, and it remained available to be completed for 3 months in total (mid-July to mid-October 2022). At the time of the study, there were 6762 veterinarians in an active professional status, according to data provided by the Portuguese Order of Veterinary Doctors. However, only veterinarians working in small animal practices in Portugal who had diagnosed and treated at least one case of feline CKD in the previous 12 months were invited to participate, after being informed that it was a voluntary and anonymous questionnaire for research purposes.

All data were collected through Google Forms and downloaded to an Excel datasheet (Microsoft Excel 2020, version 16.43.1) for further processing and descriptive statistical analysis.

The questionnaire is available as supplementary material so that its original structure can be consulted. It should be noted that not all questions present in the questionnaire were analysed in this study.

## Results

### Veterinary profile

**Figure 1:**
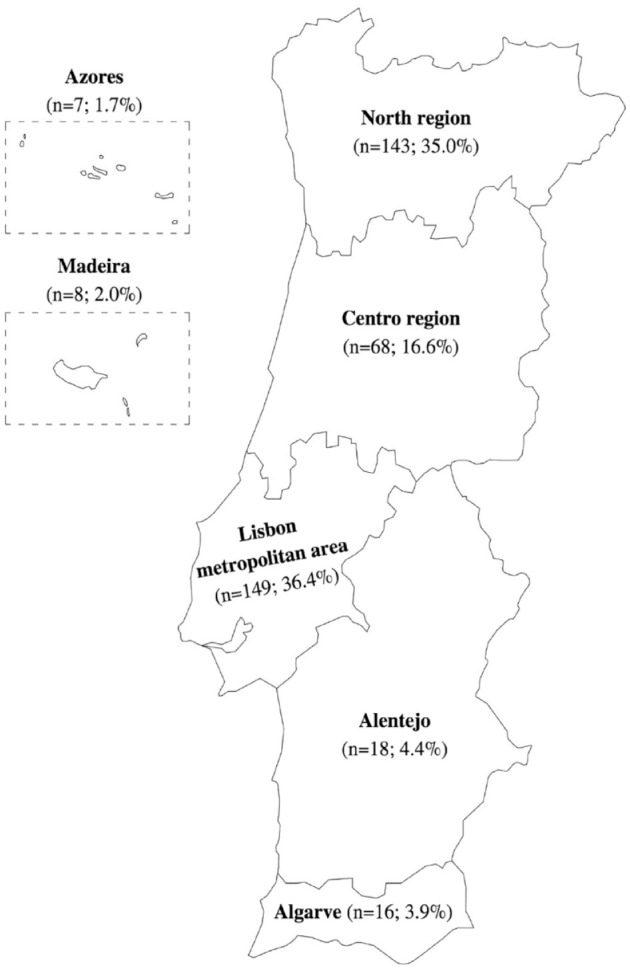
Geographical distribution of surveyed veterinary practitioners (n = 409)

A total of 409 veterinary practitioners working in Portugal responded to the questionnaire. Figure 1 shows the geographical distribution of the respondents, according to the region of the country where they carry out most of their professional activity. The median period from graduation to the time of the survey was 11 years, ranging from zero (<1 year) to 43 years.

The range of experience in feline clinical practice varied greatly in the study population as follows: <2 years (n = 36, 8.8%), 2–5 years (n = 95, 23.2%), 6–10 years (n = 93, 22.7%), 11–15 years (n = 69, 16.9%), 16–20 years (n = 56, 13.7%) or >20 years (n = 60, 14.7%). Only 2.2% (n = 9) of the surveyed veterinarians worked exclusively with feline patients. The majority (n = 365, 89.2%) worked in small animal practices (inclusive of canine and/or exotic species), with fewer being involved in livestock and/or equine medicine (n = 4, 1.0%) or in general practices (small and large animal species; n = 31, 7.6%).

### Diagnosis

Overall, approximately half of respondents (n = 219, 53.5%) diagnosed on average 2–5 cases of feline CKD per month. As expected, only work environments typically associated with larger and more resourceful facilities, such as veterinary clinics with inpatient facilities or referral veterinary hospitals, had veterinarians who diagnosed more than six cases of feline CKD per month (Table 1).

**Table 1:** Frequency of feline chronic kidney disease (CKD) diagnosis and predominant work environment, according to 409 veterinary practitioners

	Average number of CKD cases diagnosed in a month			
	⩽1	2–5	6–10	>10
Work environment				
Clinic without inpatient facilities (n = 57)	42 (73.7)	15 (26.3)	0 (0.0)	0 (0.0)
Clinic with inpatient facilities* (n = 257)	102 (39.7)	143 (55.6)	8 (3.1)	4 (1.6)
Hospital (n = 82)	7 (8.5)	55 (67.1)	16 (19.5)	4 (4.9)
Outpatient service/ambulatory care (n = 11)	5 (45.5)	6 (54.5)	0 (0.0)	0 (0.0)
Cat shelter (n = 2)	2 (100.0)	0 (0.0)	0 (0.0)	0 (0.0)
Overall	158 (38.6)	219 (53.5)	24 (5.9)	8 (2.0)

Data are n (%)

* In this study, a veterinary centre with a recovery room where animals can be accommodated for short periods of time (hours) was considered a ‘clinic with inpatient facilities’, as opposed to a hospital, where patients can remain under constant qualified surveillance for days (including overnight)

In order to support their diagnosis, in addition to the physical examination, veterinarians described performing blood tests (n = 404, 98.8%), urinalysis (n = 372, 91.0%), diagnostic imaging studies (eg, abdominal ultrasound: n = 362, 88.5%) and blood pressure measurement (n = 237, 57.9%), with 52.8% (n = 216) performing all of the studies listed above. Regarding the staging and substaging of feline CKD, 92.7% (n = 379) confirmed they were aware of the guidelines published by the IRIS.^11^ Among this subgroup, 59.1% (n = 224) measured both creatinine and SDMA, while 40.4% (n = 153) and 0.5% (n = 2) used only creatinine or only SDMA, respectively, as biomarkers. Moreover, only 19.1% (n = 78) identified that they performed SBP measurements systematically in all cats newly diagnosed with CKD (Table 2).

**Table 2:** Frequency of systolic blood pressure (SBP) measurement in cats diagnosed with chronic kidney disease (CKD), according to 409 veterinary practitioners

Frequency of SBP measurement	
Always (100% of cases)	78 (19.1)
Usually (>75% but <100% of cases)	103 (25.2)
Often (>50% but <75%)	61 (14.9)
Sometimes (>25% but ⩽50% of cases)	55 (13.4)
Rarely (>0% but ⩽25% of cases)	61 (14.9)
Never (0% of cases)	51 (12.5)

Data are n (%)

### Therapeutic approach

Regarding treatment, veterinarians were asked about how they guided the treatment prescribed to cats with CKD: 83.1% (n = 340) declared using guidelines. Within this subgroup, 93.5% (n = 318) preferentially applied those published by the IRIS.

Dietary modification to a kidney diet was recommended by almost all respondents (n = 406, 99.3%), with 73.4% (n = 298) of them advising it in all cases regardless of IRIS CKD stage, in contrast to 24.9% (n = 101) and 1.7% (n = 7) who recommended it only from IRIS CKD stages 2 and 3, respectively. Among these 406 veterinarians, the majority prescribed a commercial therapeutic diet, revealing a common preference for the combination of dry and wet diets (n = 365, 89.9%), while 9.4% (n = 38) and 0.7% (n = 3) advised only dry or only wet, respectively. A gradual diet transition was not recommended in 21.4% (n = 87) of the respondents, who usually instructed the owner to immediately change the diet. On the other hand, among the remaining respondents who advised transitioning, the majority (n = 301, 74.1%) suggested it for a period of 2 weeks or less (Table 3). The perception of the acceptance of the kidney diet varied among this subgroup, with 36.9% (n = 150) considering that the kidney diet represented <75% of the daily feeding for most of their feline patients diagnosed with CKD.

**Table 3:** Recommended time period for renal diet transition and veterinarian’s perception of renal diet acceptance, according to 406 veterinary practitioners

Recommended time period for renal diet transition (weeks)	
No transition/immediately	87 (21.4)
<1 week	144 (35.5)
1–2 weeks	157 (38.7)
3–4 weeks	12 (3.0)
5–6 weeks	2 (0.5)
7–8 weeks	1 (0.2)
>8 weeks	2 (0.5)
No response	1 (0.2)
Veterinarians’ perception of renal diet acceptance	
0 (does not tolerate the transition and maintains the conventional diet)	0 (0.0)
1 (kidney diet represents ⩽25% of the daily feeding)	11 (2.7)
2 (kidney diet represents >25% but ⩽50% of the daily feeding)	41 (10.1)
3 (kidney diet represents >50% but ⩽75% of the daily feeding)	98 (24.1)
4 (kidney diet represents >75% but <100% of the daily feeding)	175 (43.1)
5 (kidney diet represents the totality of the daily feeding)	81 (20.0)

Data are n (%)

To promote food intake, 89.5% of the surveyed veterinarians (n = 366) reported prescribing appetite stimulants, particularly oral (n = 243, 59.4%) and/or transdermal (n = 198, 48.4%) mirtazapine. The use of other drugs/nutraceuticals was also mentioned for this purpose, such as cannabidiol (n = 4), diazepam (n = 3) and cyproheptadine (n = 2).

Regarding the control of systemic hypertension in these patients, 41.8% (n = 171) of respondents preferentially prescribed a CCB, such as amlodipine, while 38.4% (n = 157) preferred an ACE inhibitor, such as enalapril or benazepril, and 19.3 % (n = 79) and 0.5% (n = 2) usually chose, respectively, an ARB, such as telmisartan, or a beta-blocker, such as propranolol or atenolol. On the other hand, to control proteinuria, most veterinary practitioners (n = 245, 59.9%) considered an ACE inhibitor as the drug of choice, while 40.1% (n = 164) preferred to use an ARB.

When asked about hydration management, 95.4% (n = 390) revealed that they used subcutaneous fluid therapy in their patients, and the most commonly administered electrolyte solution was lactated Ringer’s solution (LRS; n = 229, 56.0%) followed by a saline solution (n = 157, 38.4%).

Finally, 86.3% (n = 353) reported that they prescribed supplements as an adjuvant treatment for feline CKD, particularly phosphate binders (n = 311, 76.0%), omega-3 fatty acids (n = 106, 25.9%), multivitamin concentrates (n = 88, 21.5%), iron and B vitamins (n = 77, 18.8%), and potassium (n = 60, 14.7%).

### Monitoring

The frequency of clinical monitoring differed between what was ideally recommended by the surveyed veterinarians and what they were actually able to perform in their patients with stable CKD. Thus, 70.9% (n = 290) recommended monitoring every 2–3 months or more frequently, while in fact only 35.7% (n = 146) were able to comply with this same frequency due to the owners’ financial and time constraints (Table 4). Moreover, the tests/parameters mostly used for monitoring were creatinine and blood urea nitrogen (BUN; n = 406, 99.3%), followed by UPCR (n = 323, 79.0%), complete blood count (CBC; n = 238, 58.2%), SBP (n = 199, 48.7%), electrolyte assay (n = 194, 47.4%) and SDMA (n = 78, 19.1%).

**Table 4:** Frequency of clinical monitoring ideally recommended and actually performed, on average, in cats with chronic kidney disease (CKD) in a stable stage of the disease, according to 409 veterinary practitioners

	Clinical monitoring for a cat with CKD in a stable stage of the disease	
	Ideally recommended	Truly achievable in most cases
Frequency/periodicity		
>1 time a month	3 (0.7)	3 (0.7)
Monthly	48 (11.7)	19 (4.6)
Every 2–3 months	239 (58.4)	124 (30.3)
Every 4–5 months	50 (12.2)	54 (13.2)
Every 6 months	64 (15.6)	107 (26.2)
Every 7 or more months	1 (0.2)	46 (11.2)
Only when clinical signs reappear	4 (1.0)	56 (13.7)

Data are n (%)

## Discussion

The results indicate that most veterinarians who completed this questionnaire-based survey use a combination of complementary tests for the diagnosis of CKD in cats, including blood and urine tests and abdominal ultrasound. In clinical practice, blood analysis is one of the first tests performed on these patients when CKD is suspected, allowing the detection of azotaemia, through the increase of serum levels of creatinine and BUN.^8^ In addition, as there are often electrolyte disturbances, such as hypokalaemia and hyperphosphataemia, an evaluation of the electrolyte status should also always be recommended.^17^ A CBC provides important information to the clinician in relation to the development of anaemia, including evidence of a decreased erythrocyte count, haematocrit and haemoglobin concentrations, which is a common finding in cats with IRIS CKD stages 3 and 4.^18,19^ Although in this study, the blood parameters most requested by the responding veterinarians were not individually evaluated, it is routine practice for renal panels performed at Portuguese veterinary diagnostic laboratories to include CBC, BUN, creatinine, albumin, total protein, sodium, chloride, potassium, phosphorus and calcium. The second most common diagnostic test was a urinalysis, which was reportedly performed by 91% of respondents. The questionnaire did not require respondents to indicate the specific urinary parameters that they would usually evaluate. However, in Portuguese veterinary diagnostic laboratories, a basic urinalysis would routinely include a USG, a dipstick test and sediment evaluation, with the latter being important to exclude post-renal causes of proteinuria.^20^ With regard to renal proteinuria, the UPCR is the parameter of choice, often being requested in association with the aforementioned analysis.^21^ Despite the diagnostic relevance of performing a urinalysis, a questionnaire-based study addressed to owners of cats with CKD reported that in 23% of cases the veterinarian responsible for their cat’s care had never performed a urinalysis.^22^ Fortunately, in the current surveyed population, a urinalysis was part of the diagnostic and monitoring plan for most of the responding veterinarians, likely supported by urinalysis being a routine laboratory practice in Portugal.

Most respondents expressed awareness and knowledge of the IRIS staging guidelines, whose correct use is of critical importance to ensure that any therapeutic interventions are appropriate to the respective disease stage and therefore maximise the potential to reduce progression of the disease over time.^11,13^ Moreover, the application of the IRIS staging system is crucial for discussing the prognosis with the owners, since the IRIS CKD stage at diagnosis is considered a significant predictor of the patient’s survival.^18,23^ Current IRIS guidelines recommend measuring both creatinine and SDMA for an appropriate CKD staging.^11^ SDMA has been considered a more sensitive marker for the detection of CKD,^12^ which allows an earlier diagnosis and facilitates clinical intervention in the early stages of the disease, creating the potential to improve prognosis. Furthermore, SDMA concentration is not influenced by lean body mass, which in patients with muscle loss may be important for a more accurate staging, since the use of the creatinine values alone may underestimate the IRIS CKD stage.^10^ Nevertheless, elevated SDMA concentrations may also be present in non-renal patients and in association with normal serum creatinine concentrations, namely in some cats diagnosed with lymphoma, as recently described.^24^ In the present study, despite more than half of the respondents stating that they use both biomarkers for diagnosing and staging CKD, approximately 40% continue to measure only creatinine for this purpose. One presumable justification could be the higher financial cost associated with the SDMA measurement, which often has to be requested separately, as it is not yet part of most commercial renal analytical panels in Portugal.

An SBP measurement is another important procedure when diagnosing and monitoring cats with CKD, as systemic hypertension has been frequently associated with CKD.^25–27^ According to IRIS guidelines, feline patients are substaged based on SBP values, which allows the clinician to determine the risk of target organ damage.^11^ Despite this fact, 27.4% of responding veterinarians admitted never measuring SBP or performing it only rarely, which is in line with some published studies that failed to measure SBP during follow-up in cats with kidney disease.^18,28,29^ These data suggest that substaging was not being adequately completed by most respondents, highlighting the fact that hypertension has probably been underdiagnosed in this type of population, which increases the risk of clinical complications not only in the kidneys, but also in the eyes, brain, and heart and vasculature.^25^ There could be several reasons why the routine measurement of blood pressure is not more regularly and systematically performed, such as the absence of Doppler or oscillometric devices in the work environment, a lack of knowledge of the clinical importance of measuring blood pressure, or apparent difficulty or unwillingness to measure blood pressure in conscious cats. Regarding the last issue, an acclimatisation period, careful and gentle animal handling and restraint, and the use of the coccygeal artery for SBP measurement have all been described as strategies that can increase cats’ tolerance for this examination.^30–32^ According to the International Society of Feline Medicine (ISFM) Consensus Guidelines on the Diagnosis and Management of Hypertension in Cats, cats diagnosed with CKD should have their SBP monitored as soon as they are diagnosed and at least every 3–6 months thereafter.^25^ Some authors have even suggested that cats with higher SBP at the time of diagnosis should be monitored more regularly, as they are more likely to manifest clinically relevant changes resulting from hypertension.^27^

With regard to the therapeutic approach, dietary intervention is a key aspect, and a transition to a good-quality commercial renal diet has been recommended for all cats in IRIS CKD stages 2–4.^8,13^ For its formulation, concentrations of protein, phosphorus, sodium, potassium, vitamin D, calcium and omega-3 fatty acids have been mainly considered.^33^ In addition to helping delay and/or stabilise CKD,^33^ therapeutic renal diets have also been associated with a significant decrease in uraemic episodes and CKD-related deaths,^20^ a significant increase in the survival of these patients compared with those that remain on a maintenance diet^15^ and an improvement in patients’ quality of life by increasing their energy levels, interaction and vitality.^16^ Almost all respondents reported prescribing a renal therapeutic diet, with a preference for a combination of dry and wet/canned foods, which is in line with findings described by owners in other studies.^22^ However, a significant percentage of veterinarians considered that the acceptance of the renal diet was only partial in most of their patients. From the authors’ clinical experience, the crucial factor for the acceptance of the renal diet is not its palatability, but the moment at which it is introduced. For example, Fritsch et al^16^ described that the transition to renal food was well accepted by most animals (93.8%), with the respective owners reporting that their cats liked it as much as or more than their previous diet. In the present study and considering the answers provided, it can be hypothesised that failure to transition to a therapeutic renal diet may be the consequence of respondents using a renal diet irrespective of IRIS CKD stage and without an adequate transition period. Renal diets should ideally be prescribed in early IRIS CKD stage 2 to maximise acceptance, as in later stages (IRIS CKD stages 3 and 4) extrarenal clinical signs, such as nausea and vomiting, can result in inappetence and food aversion.^13^ To ensure greater success, the transition should be slow, lasting 4–8 weeks in total. During this time, the proportion of previous food should be gradually decreased in favour of the therapeutic renal diet.^8^ Another aspect that has been described for the failure of nutritional management in some patients is the owner’s lack of compliance in providing an appropriate renal diet for their cat.^15,16^ In fact, many owners may not be receiving adequate instructions from the veterinarian on how to introduce this therapeutic diet. This hypothesis was not investigated in this study, but Markovich et al^22^ reported that 34.0% of the owners questioned followed dietary recommendations for their cats with CKD, based on information collected from the internet, pet stores, friends and breeders. These sources of information can be problematic as most are not directly controlled by professionals with the expertise required for animal nutritional advice, which can lead to inappropriate dietary interventions. Moreover, most veterinarians in this study prescribed oral and/or transdermal mirtazapine to these patients, in accordance with IRIS recommendations that suggest the adjunctive use of appetite stimulants as part of the nutritional management, particularly in cats with CKD with inappetence.^13^

For cats with CKD where systemic hypertension and/or persistent proteinuria is identified, it is recommended that specific anti-hypertensive and anti-proteinuric therapy should be prescribed. In the current study, amlodipine was the drug most frequently prescribed by the surveyed veterinarians for the treatment of systemic hypertension. This is in line with guidelines for the use of amlodipine and current licensing data.^25,34^ The efficacy and safety of amlodipine as an anti-hypertensive agent has been described in several clinical trials performed in cats with CKD.^5,20,27^ More recently, telmisartan, an ARB first used to control proteinuria, was also licensed as an anti-hypertensive agent at a dose of 2 mg/kg per day, due to its significant efficacy and good tolerability.^35,36^ Alternative drugs, such as an ACE inhibitor or a beta-blocker, which were chosen to control hypertension in almost 40% of respondents, are not currently recommended as a first and only choice given their limited effect on lowering SBP.^34^ To control renal proteinuria, ACE inhibitors, namely benazepril, have been used as preferred drugs, which reflects the common knowledge of the studied population regarding their anti-proteinuric effect.^28,29^ In fact, benazepril at a dose of 0.5–1 mg/kg per day has been recommended for proteinuric feline patients given its significant effect in reducing the UPCR.^28,29,37^ Furthermore, 40.1% of respondents mentioned opting for telmisartan, which was previously found to be equally or even more effective in reducing proteinuria.^38^

Fluid therapy has been recommended in cats with renal disease to correct clinical dehydration and electrolyte and acid–base disturbances. Fluids can be provided by either intravenous or subcutaneous administration, depending on the patient’s needs.^8,13^ The present results showed that the prescription of subcutaneous fluid therapy in feline patients with CKD is a common practice among responding veterinarians, as it is in other countries.^22,39^ This supportive therapy is often performed in a clinical environment, but it is increasingly common to teach owners how to do administer subcutaneous fluids at home in order to avoid unnecessary visits to the clinic, reducing patient stress and potentially increasing long-term owner compliance. In fact, Cooley et al^39^ showed that most owners are comfortable providing subcutaneous fluids, considering it to be easy, low-stress and tolerable for their cat. LRS was the electrolyte solution most often administered by respondents, which is in line with previous findings.^22^ This solution, particularly half-strength LRS, has been considered a suitable choice because it has a lower sodium and higher potassium content compared with saline solution (0.9% sodium chloride).^8,17^ However, it should be noted that fluid therapy is not always necessary, as some patients with compensated CKD may not need it if they are stable and adequately hydrated.^17^ Fluids should be given with caution, particularly in cats with concurrent cardiac disease due to the risk of contributing to the development of congestive heart failure in these patients.^40^

In addition, despite the finding that transition to a therapeutic renal diet was not always successful, many veterinarians were prescribing phosphate binders. This is controversial since the use of a phosphate-binder with a maintenance diet is unlikely to achieve the required phosphate regulation, and concurrent use with a phosphate-restricted renal diet is recommended.^14^ Nevertheless, when hyperphosphataemia persists 1 month after starting the renal diet or when the renal diet is not accepted by the patient, the administration of a phosphate binder has been advised.^8,41^ Commonly used phosphate binders, either aluminium hydroxide or calcium carbonate (starting dose: 30–60 mg/kg/day divided into several meals), are always administered with food in order to facilitate intestinal phosphate binding and excretion.^13^

In relation to other nutraceuticals, the frequency of prescription was much lower. About one-quarter of veterinarians revealed that they prescribe omega-3 fatty acid supplementation to their patients with CKD. Although the evidence of its therapeutic effect in feline CKD is still lacking, the results obtained in a previous retrospective study^42^ suggested its potential benefit in the survival of these patients, which has been thought to result from their ability to reduce inflammation by promoting the production of less inflammatory eicosanoids and pro-resolving mediators^43^ and to increase the bioavailability of nitric oxide,^44^ improving kidney function and slowing the progression of the disease. Moreover, the administration of multivitamin concentrates and supplementation with iron and B vitamins were also described, which are recommended due to their antioxidant potential and to support erythropoiesis in patients with anaemia, respectively.^8^ These products must be prescribed in combination with other therapies, such as the use of erythrocyte-stimulating agents.^19^ Finally, a small percentage of veterinarians reported using potassium supplements, which, according to the literature, should be administered where there is evidence of hypokalaemia (serum potassium concentrations <3.5 mmol/l), an electrolyte imbalance that results mainly from increased urinary excretion of potassium in these animals.^8,15^ Thus, apart from phosphate binders, adjuvant therapy with supplements was not a common practice by most clinicians. One of the main reasons given for the low percentage of cats taking supplements is their lack of palatability.^9^

Once diagnosed and treatment initiated, CKD must be monitored for the rest of the cat’s life. According to the ISFM Consensus Guidelines on the Diagnosis and Management of Feline Chronic Kidney Disease, stable cats should be monitored at least every 3–6 months, in order to assess for progression and need to restage. This should be based on a complete physical examination, blood and urine analysis, SBP measurement and imaging reassessment.^8^ However, one-quarter of veterinarians mentioned not being able to maintain this periodicity, with re-examinations ultimately occurring every 7 months or more, or only when clinical signs relapsed. In addition, there was a great disparity between the ideal monitoring interval recommended by veterinarians and the interval achieved. Further work should focus on understanding the main causes of this lack of compliance. Some possible factors may be financial restrictions, time constraints, negative experiences at the clinic/hospital or geographic distance, for example. It is important to address this issue, raising owners’ awareness to comply more strictly with the monitoring plan due to its impact on their cats’ prognosis. In this monitoring process, the second most frequently evaluated parameter, after serum creatinine and BUN concentrations, was the UPCR, showing that most veterinarians know the importance of controlling proteinuria, a poor prognostic factor with a significantly negative impact on patient survival.^9,18,30^

The present study has some limitations that the authors would like to emphasise. First, the potential bias associated with filling out the questionnaire cannot be neglected, since completion ultimately depended on the goodwill of the veterinarians. Thus, clinicians with greater interest or knowledge in feline medicine, particularly nephrology, as well as those who value scientific endeavours to improve evidence-based clinical practice, may be more over-represented compared with the general veterinarian population. Second, the questionnaire design lacked specificity in some areas, such as, specific testing parameters, such that responses were at times generalised. Even so, the total number of responses was considered satisfactory in comparison with other studies addressed to Portuguese veterinary practitioners,^45,46^ and we believe that the results obtained represent the approach of the surveyed veterinarians for most of their cases of feline CKD.

## Conclusions

This study showed that most survey respondents use international guidelines for the clinical management of cats diagnosed with CKD, although the substaging is not being properly carried out. In fact, SBP measurement is still not a systematic practice, which compromises the correct substaging of these patients and prevents the proper diagnosis of systemic hypertension, which consequently increases the risk of target organ damage. In addition, these findings showed that the dietary intervention must be refined, as the renal diet is being introduced indiscriminately and without respect for the ideal gradual transition, possibly limiting its acceptance. Finally, the reported monitoring frequency showed that it is necessary to investigate the potential factors responsible for the owners’ lack of compliance with these recommendations, in order to create solutions that promote more regular reassessments, which is crucial for a more successful clinical management of feline CKD.

## Supplemental Material

Supplemental Material Click here for additional data file. Online questionnaire provided to all small animal veterinarians working in Portugal: ‘Clinical management of feline chronic kidney disease in Portugal’ (original questionnaire language: Portuguese).
