# Supplementary material for: Clinical management of feline chronic kidney disease in Portugal: a questionnaire-based study
Source: J Feline Med Surg. 2023 Nov 21;25(11):1098612X231206125. doi: 10.1177/1098612X231206125 (PMC10811999; doi:10.1177/1098612X231206125)
Supplement: Supplemental Material [file sj-docx-1-jfm-10.1177_1098612X231206125.docx]

**SUPPLEMENTAL MATERIAL**

Online questionnaire provided to all small animal veterinarians working in Portugal: "Clinical management of feline chronic kidney disease in Portugal" (original questionnaire language: Portuguese)

**Clinical management of feline chronic kidney disease in Portugal**

This questionnaire aims to collect information on how Portuguese veterinarians are currently performing the clinical management of feline chronic kidney disease (CKD), particularly with regard to its diagnosis and treatment. For this reason, it is intended for veterinarians working in small animal practice in Portugal (mainland and autonomous regions) and who have diagnosed and treated at least one case of CKD in the last year.

The questionnaire consists of 30 questions divided into 3 parts (“Veterinarian profile”, “Diagnosis and monitoring” and “Therapeutic approach”) and it will take about 10 minutes to answer. It should be noted that the questionnaire is voluntary, and that no private personal information is required, so we ask you to answer honestly, based on your experience, so that we can obtain valid and reliable results that help us to draw an accurate “picture” of our country on this topic. If you have any questions regarding our privacy policy, we suggest that you consult the privacy statement of the University of Trás-os-Montes and Alto Douro (www.utad.pt/app-privacy-policy).

The results of this study will be treated with the utmost confidentiality with a view to producing works for public dissemination, namely in the form of a scientific article.

In case of doubt while filling out the questionnaire, please contact us via email to [tomas.rodrigues.magalhaes@gmail.com](mailto:tomas.rodrigues.magalhaes@gmail.com), so that we can better clarify it.

If you voluntarily agree to participate in this study and authorize the use of your answers for scientific purposes, proceed with filling it out.

We thank you in advance for your collaboration and for the time you will spend answering our questions.

Abbreviations:

ACEI – Angiotensin-converting enzyme inhibitor

ARB – Angiotensin receptor blocker

CKD – Chronic kidney disease

IRIS – International Renal Interest Society

SDMA – Symmetrical dimethylarginine (SDMA)

VETERINARIAN PROFILE

1. What is the highest academic degree you have completed in veterinary medicine? (select only one option)
2. Doctor in Veterinary Medicine (DVM) (pre-Bologna).
3. Doctor in Veterinary Medicine (DVM) (pre-Bologna) + Master of Science (MSc)
4. Master in Science (MSci) (post-Bologna)
5. Doctor of Philosophy (PhD).
6. In which university did you get the diploma to practice veterinary medicine? (select only one option)
7. University of Lisbon.
8. University of Trás-os-Montes and Alto Douro.
9. University of Porto.
10. University of Évora.
11. Vasco da Gama University School.
12. Lusófona University of Lisbon.
13. Other. Please indicate: ______.
14. Please indicate in which year you graduated in veterinary medicine: ______.
15. Apart from your academic background, do you have any certified advanced training in veterinary medicine? (select only one option)
    1. Yes, I have a General Practitioner Certificate (GPCert) and/or a General Practitioner Advanced Certificate (GPAdvCert).
    2. Yes, I have a Postgraduate Certificate (PgC).
    3. Yes, I’m an EBVS^®^ European Veterinary Specialist.
    4. No.
    5. Other. Please indicate: ______.
16. What is your experience in feline clinical practice? (select only one option)
17. Less than 2 years.
18. 2 to 5 years.
19. 6 to 10 years.
20. 11 to 15 years.
21. 16 to 20 years.
22. More than 20 years.
23. What is your predominant work environment? (select only one option)
24. Clinic without inpatient facilities.
25. Clinic with inpatient facilities.
26. Hospital.
27. Outpatient service/ Ambulatory care.
28. Cat shelter.
29. Regarding clinical practice, are you dedicated exclusively to feline medicine? (select only one option)
30. Yes.
31. No, I also practice canine and/or exotic species medicine.
32. No, I also practice livestock and/or equine medicine.
33. No, I practice a more general medicine that simultaneously includes species from b) and c).
34. In which region of the country do you carry out most of your professional activity? (select only one option)
35. North region.
36. Centro region.
37. Lisbon metropolitan area.
38. Alentejo.
39. Algarve.
40. Madeira.
41. Azores.

DIAGNOSIS AND MONITORING

1. How many cases of feline chronic kidney disease (CKD) do you diagnose, on average, per month? (select only one option)
2. 1 case or less.
3. 2 to 5 cases.
4. 6 to 10 cases.
5. More than 10 cases.
6. In addition to the physical examination, what other tests generally support your diagnosis of CKD in cats? (select one or more options)
7. Blood tests.
8. Urine analysis.
9. Imaging exams (e.g., abdominal ultrasound).
10. Blood pressure measurment.
11. I do not perform additional tests since I make my diagnosis exclusively through anamnesis and physical examination.
12. Are you aware of the guidelines published by the International Renal Interest Society (iris-kidney.com) for the staging and substaging of CKD? (select only one option)
13. Yes.
14. No.
15. If you answered yes to the previous question, what biomarkers do you use for staging? (select only one option)
16. Only creatinine.
17. Only symmetric dimethylarginine (SDMA).
18. Creatinine and SDMA.
19. Do you measure systolic blood pressure in cats you diagnose with CKD? (select only one option)
20. Always (100% of cases).
21. Usually (> 75% but < 100% of cases).
22. Often (> 50% but ≤ 75% of cases).
23. Sometimes (> 25% but ≤ 50% of cases).
24. Rarely (> 0% but ≤ 25% of cases).
25. Never (0% of cases).
26. Which tests/ parameters do you routinely use to monitor feline CKD? (select one or more options)
27. Complete blood count (CBC).
28. Urea and creatinine.
29. SDMA.
30. Electrolyte test.
31. Urinary protein-to-creatinine (UPC) ratio.
32. Blood pressure.
33. How often, on average, do you ideally recommend monitoring a cat with CKD at a stable stage of the disease? (select only one option)
34. More than once a month.
35. Monthly.
36. Every 2 to 3 months.
37. Every 4 to 5 months.
38. Every 6 months.
39. Every 7 or more months.
40. Only when clinical signs reappear.
41. In line with the previous question, how often, on average, are you able to actually monitor a cat with CKD in a stable stage of the disease, considering not only your medical recommendation, but also the owner's economic and time constraints? (select only one option)
42. More than once a month.
43. Monthly.
44. Every 2 to 3 months.
45. Every 4 to 5 months.
46. Every 6 months.
47. Every 7 or more months.
48. Only when clinical signs reappear.

THERAPEUTIC APPROACH

1. Do you use guidelines for the treatment you prescribe for cats with CKD? (select only one option)
2. Yes.
3. No.
4. If you answered yes to the previous question, which guidelines do you prefer to use?
5. International Society of Feline Medicine (ISFM) Consensus Guidelines.
6. International Renal Interest Society (IRIS) Guidelines.
7. Other. Please indicate: ______.
8. Do you recommend switching to a kidney diet for all cats you diagnose with CKD? (select only one option)
9. Yes, all.
10. No, only from IRIS stage 2.
11. No, only from IRIS stage 3.
12. No, only in IRIS stage 4.
13. No, never. (Proceed to question 24)
14. Which commercial therapeutic diet, intended for cats with renal disease, do you most recommend? (select only one option)
15. Hill’s™ Prescription Diet™ Feline k/d™.
16. Royal Canin Veterinary Diet^®^ Renal™ Cat/ Renal Special™ Cat.
17. Purina^®^ Pro Plan^®^ Veterinary Diets Feline NF St/Ox Renal Function™.
18. Advance Veterinary Diets^®^ Renal Failure Feline.
19. Specific™ Cat Kidney Support (FKD/FKW).
20. Virbac Veterinary HPM^®^ K1 Cat Kidney Support.
21. Other. Please indicate: ______.
22. Which type of kidney diet do you usually prescribe? (select only one option)
23. Dry.
24. Wet.
25. Dry and wet.
26. How long do you normally recommend transitioning to a kidney diet? (select only one option)
27. I do not recommend a transition period, as I suggest an immediate dietary change.
28. 1 to 2 weeks.
29. 3 to 4 weeks.
30. 5 to 6 weeks.
31. 7 to 8 weeks.
32. More than 8 weeks.
33. What is your perception regarding the acceptance of the kidney diet by the majority of your feline patients, on a scale of 0 to 5? (select only one option)
34. 0 (does not tolerate the transition and maintains the conventional diet)
35. 1 (kidney diet represents ≤ 25% of the daily food intake)
36. 2 (kidney diet represents > 25%, but ≤ 50% of the daily food intake).
37. 3 (kidney diet represents > 50% but ≤ 75% of the daily food intake).
38. 4 (kidney diet > 75% but < 100% of the daily food intake).
39. 5 (kidney diet represents the totality of the daily food intake).
40. Do you often prescribe appetite stimulants to promote food intake? (select one or more options)
41. Yes, oral mirtazapine.
42. Yes, transdermal mirtazapine.
43. Yes, another appetite stimulant. Please indicate: ______.
44. No, I don't use appetite stimulants.
45. Which drug do you prefer to use to control systemic hypertension in feline patients with CKD? (select only one option)
46. Angiotensin-converting enzyme inhibitor (ACEI; e.g., enalapril and benazepril).
47. Angiotensin receptor blocker (ARB; e.g, telmisartan).
48. Calcium channel blocker (ex: amlodipine).
49. Beta-blocker (e.g., propranolol and atenolol).
50. Other. Please indicate: ______.
51. Which drug do you prefer to use to control proteinuria in feline patients with CKD? (select only one option)
    1. Angiotensin-converting enzyme inhibitor (ACEI; e.g., enalapril and benazepril).
    2. Angiotensin receptor blocker (ARB; e.g, telmisartan).
    3. Other. Please indicate: ______.
52. Which electrolyte solution do you most often use in subcutaneous fluid therapy? (select only one option)
53. Lactated Ringer’s solution.
54. Saline solution.
55. Other. Please indicate: ______.
56. None, I do not use subcutaneous fluid therapy in my patients.
57. Do you prescribe supplements as an adjuvant treatment for feline CKD? (select only one option)
58. Yes.
59. No.
60. If you answered yes to the previous question, which nutraceuticals do you most often prescribe (select one or more options)
61. Phosphate binders.
62. Omega-3 fatty acids.
63. Iron and B vitamins.
64. Potassium.
65. Multi vitamin concentrates.
66. Other. Please indicate: ______.
67. Finally, if you have any comments to add regarding the way you perform the clinical management of feline CKD, in addition to what has already been addressed in the questionnaire, feel free to use the space below.
